# Fishy factors: recognizing biological variation and its implications for fish immuno(eco)toxicology research

**DOI:** 10.1093/etojnl/vgae085

**Published:** 2025-01-06

**Authors:** Rashidat O Jimoh, Cheyenne R Smith, Vicki S Blazer, Jone Corrales, Natacha S Hogan, Maria L Rodgers, Catherine Wise, Marlo K Sellin Jeffries

**Affiliations:** Department of Biology, Texas Christian University, Fort Worth, TX, United States; Eastern Ecological Science Center -Leetown Research Laboratory, U.S. Geological Survey, Kearneysville, WV, United States; Eastern Ecological Science Center -Leetown Research Laboratory, U.S. Geological Survey, Kearneysville, WV, United States; Office of Chemical Safety and Pollution Prevention, U.S. Environmental Protection Agency, Washington, DC, United States; Toxicology Centre, University of Saskatchewan, Saskatoon, Canada; Department of Animal and Poultry Science, College of Agriculture and Bioresources, University of Saskatchewan, Saskatoon, Canada; Department of Biological Sciences, North Carolina State University, Center for Marine Sciences and Technology, Morehead City, NC, United States; Department of Biology, Texas Christian University, Fort Worth, TX, United States; Department of Biology, Texas Christian University, Fort Worth, TX, United States

Focus articles are part of a regular series intended to sharpen understanding of current and emerging topics of interest to the scientific community.

## Introduction

The immune system, which protects organisms from infectious and noninfectious diseases, is sensitive to toxicants. In some instances, chemical-induced immune dysfunction, resulting from either direct or indirect effects on key components of the immune system, can alter the ability of an organism to appropriately respond to pathogens, leading to a heightened risk of disease and death (Rehberger et al., 2017; Rice et al., 2024). Alternatively, chemical exposures can stimulate the immune system, leading to autoimmunity or hypersensitivity. In some cases, these effects may occur at concentrations well below thresholds for acute or chronic toxicity. Although immunotoxicity can affect all species, fish may be particularly vulnerable given their direct and constant interaction with water, a medium that is prone to contamination by a broad range of chemical classes and can harbor pathogens such as bacteria, viruses, and parasites. As such, the assessment of immunotoxicity in fish has become increasingly important for the protection of both cultured and wild populations.

The importance of this work is underscored by the ecosystem, economic, and cultural services provided by fish (Holmlund & Hammer, 1999). Fish are key components of food webs, regulate nutrient recycling and substrate distribution, and contribute to ecosystem resilience and diversity. From economic and cultural perspectives, fish represent a vital nutrient source, as they provide 17% of animal protein intake globally, represent over $51 billion in international trade value annually, and provide recreational opportunities for 200–700 million people annually (Food and Agriculture Organization, 2020). Furthermore, the fisheries sector promotes socioeconomic growth, reduces poverty, and improves the living conditions of vulnerable communities (Chan et al., 2019). Thus, mortality events due to infectious diseases in both captive and wild populations can have severe ecological, economic, and cultural consequences (Kataoka & Kashiwada, 2021; Lafferty et al., 2015). Fish are also useful surrogates for predicting the impacts of chemical exposures on other vertebrate organisms. Their immunotoxic responses can enhance our ability to predict chemical exposure impacts on immune function across a wide range of vertebrate taxa. Hence, understanding the role of toxicants and other environmental stressors on immune system function is necessary to develop effective management approaches that not only enhance the health of cultured fish and protect wild fish populations, but also provide broad protection to aquatic resources and the associated ecosystem services.

**Figure 1.**
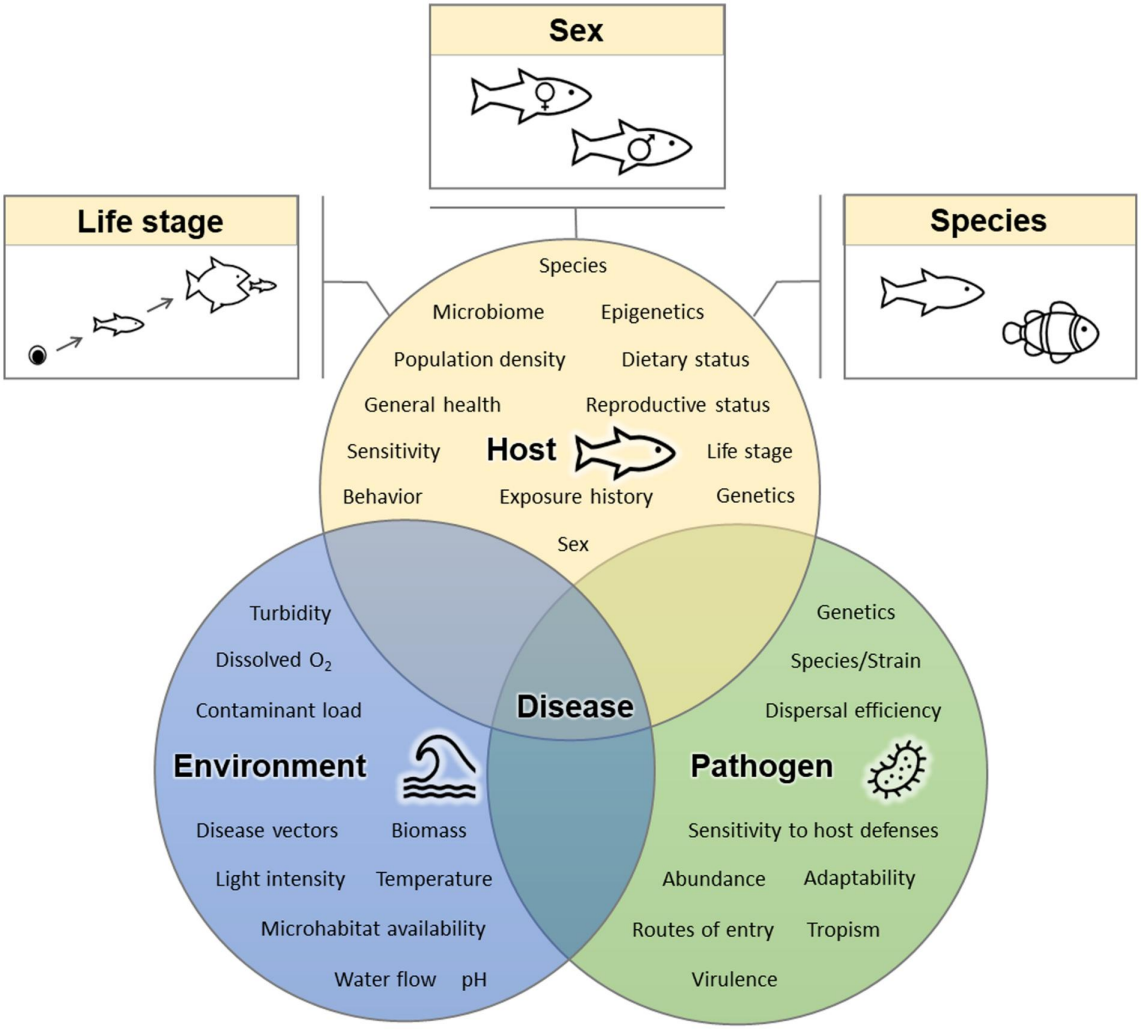
A conceptual model, based on the disease triangle originally developed by McNew (1960), showing how the host, pathogen, and environment interact may interact to induce disease. There are numerous factors that may influence environmental conditions that promote disease, host susceptibility, and pathogen success. A variety of these factors are provided and the three “fishy factors” of interest in this article, life stage, sex, and species, are indicated.

Despite a critical need to understand how exposure to contaminants affects fish immunity and disease occurrence, immunotoxicity research on fish has lagged behind other topics, such as endocrine disruption. This is due, in part, to a general lack of understanding regarding the specific factors that influence immune function and disease occurrence in fish. Accurately predicting the likelihood and severity of disease requires careful consideration of the interactions among three key factors: the host, the pathogen, and the environment (each of which are components of the “disease triangle” developed by McNew [1960] and more recently reviewed by Scholthof [2007]). As depicted in Figure 1, each of these factors encompasses a complex suite of parameters. Environmental factors include both abiotic (e.g., contaminant load, temperature, dissolved oxygen levels, etc.) and biotic (e.g., presence of disease vectors, biomass of host organisms, etc.) parameters. Pathogen-associated factors include a multitude of biotic parameters such as species, abundance, virulence, and adaptability (See Bricknell, 2017 for a review of fish pathogens). Similarly, host-associated factors encompass biotic parameters including species, population density, and sensitivity. Although there are a multitude of environment, pathogen, and host variables that can influence the immunotoxic responses of fish, three key intrinsic sources of variation associated with the host will be explored here: life stage, sex, and species. We have specifically chosen these “fishy factors” because they may be accounted for a priori in laboratory studies (via appropriate experimental design) and are often easy to discern and account for post hoc in field studies.

## Life stage-dependent differences in immunity and immunotoxic responses

Fish progress through many different and unique life stages, including embryo, eleutheroembryo, larva, juvenile, and adult. Development of the immune system depends on a cascade of carefully coordinated events originating during embryogenesis and continuing into adulthood. The specific ontogeny of immune system varies among fish species; however, some aspects of immune system development can be generalized across many teleosts (reviewed by Zapata et al., 2006; Buchmann et al. 2024). Fish embryos possess maternally derived proteins (e.g., IgM, lysozyme, complement components, etc.) and mRNAs (e.g., antimicrobial peptides, complement components, etc.) which, in combination with the physical barrier of the chorion and associated membranes, provide temporary protection beginning at fertilization (Zhang et al., 2013). Prior to hatch, embryos begin to express immune genes, exhibit signs of head kidney and thymus formation, and produce key immune cells including lymphocytes and myeloid cells (Buchmann et al., 2024; Zapata et al., 2006). Shortly after hatch, when eleutheroembryos enter aquatic environments and lose the barrier function of the chorion, the expression of both innate and adaptive immune genes rises substantially and the presence of a well-developed thymus and head kidney become apparent, indicating continued rapid development of immune system functionality. As development progresses, the spleen forms, adaptive immune gene expression continues to rise, immune cells mature, and immune tissues become densely populated with lymphocytes. Given the pattern of immune system development, fish are highly reliant on surface barrier protection and innate immune factors during early-life stages and use a combination of both innate and adaptive immune responses later in life. As such, life stage influences susceptibility to pathogen infection and disease outcome. In general, early-life-stage fish are often considered more susceptible to disease than adult fish due to their reduced ability to mount adaptive immune responses to pathogens. However, there is evidence to suggest that fish embryos and eleutheroembryos may be less susceptible to some bacterial and viral infections relative to their older counterparts due to strong physical barriers (e.g., chorion, mucosal layers, etc.; Buchmann et al., 2024). Thus, it appears that larval and juvenile fish may be more susceptible to some pathogens than embryos, eleutheroembryos, and adults.

As is the case with pathogens, fish also exhibit differential sensitivities to environmental contaminants across life stages. Exposures to contaminants can produce either activational or organizational effects (Guillette et al., 1995). Activational effects, which may occur in response to contaminant exposures at any life stage, are temporary, reversible, and often characterized by changes in gene expression patterns, protein levels, or enzymatic activity. In contrast, organizational effects, which typically occur in response to early-life-stage chemical exposures, are long-term, irreversible, and characterized by cell- or tissue-level structural changes that alter physiological function. Early-life-stage exposures to contaminants may alter the development of immune system components, leading to permanent alterations in immune function and disease resistance. In fact, a previous study showed that Chinook salmon (*Oncorhynchus tshawytscha*) subjected to short-term exposures to o, p ’-dichlorodiphenyldichloroethylene as embryos and eleutheroembryos displayed reduced leukocyte blastogenesis 1 year after the initial exposure indicating long-term alterations in humoral immunity (Milston et al., 2003). Another study, by Thornton-Hampton et al. (2021), demonstrated that developmental exposures to the thyroid-hormone disruptor propylthiouracil caused long-term alterations in the phagocytic activity of head kidney isolates from fathead minnows (*Pimephales promelas*). The findings of these studies suggest that early-life-stage fish are susceptible to permanent alterations in immune function in response to chemical exposures. Despite this, as reported by Rehberger et al. (2017), most immunotoxicity studies (61% of the 241 studies analyzed) used “juvenile” fish and only 9% featured the use of embryos. In light of this and the potential for organizational effects, which are arguably more severe than activational effects, there is a need for additional studies aimed at identifying windows of sensitivity for long-term immune disruption in fish and understanding the long-term consequences of developmental immunotoxicity on immunity and disease occurrence.

## Sex-based differences in immunity and immunotoxic responses

Organism sex is a key factor influencing a variety of physiological processes, including immunity. Various factors, including X-linked genes, hormones, and life history strategies, contribute to distinct sex differences in immune response (Fish, 2008). In general, females are considered to initiate more effective innate and adaptive immune responses than males across a wide variety of vertebrate species. Although studies investigating sex-specific immune differences among fish are sparse, there is evidence that such differences exist. For example, in a laboratory exposure study, female fathead minnows had higher rates of survival following a bacterial exposure than their male counterparts (Thornton et al., 2018). Conversely, a weaker antiviral response (i.e., lower expression of interferon) and increased mortality were reported in female zebrafish (*Danio rerio*; Lu et al., 2022). In mammals, females are more prone to autoimmunity and hypersensitivity reactions (Fish, 2008), but few studies have investigated the development of autoimmunity or hypersensitivity reactions in fish, so the extent to which sex influences such responses remains unclear. Nevertheless, it is important to recognize that many of the immune cells and signaling molecules associated with these processes in mammals are also present in teleosts, suggesting that sex-specific differences may occur.

Across vertebrates, sex-specific differences in immunity are thought to stem primarily from differences in the expression of sex chromosome–encoded genes and circulating levels of sex hormones (Lotter & Altfeld, 2019). Evidence from mammalian studies suggests that X-linked genes code for proteins involved in immunity, including pattern recognition and toll-like receptors (Schurz et al., 2019). The extent to which this holds true in fish has yet to be determined, and the diverse nature of sex determination in fish suggests that the differential expression of sex-linked genes is likely to be species dependent. With regard to circulating sex hormones, much of what is known regarding the impacts of sex hormones on fish immunity is inferred based on differences in immunocompetence during different phases of the reproductive cycle, which are characterized by notable changes in circulating levels of sex-steroid hormones (Milla et al., 2011). Androgens are generally thought to suppress some key immune processes, including respiratory burst, phagocytosis, lymphocyte proliferation, and IgM production, although there are contradictory reports (reviewed by Chuphal et al., 2023). The impacts of estrogens are more varied, as some studies have shown that estrogens enhance the immune response and disease resistance, whereas others indicate the opposite, suggesting effects are both species specific and response specific (Chuphal et al., 2023). In addition to sex steroid hormones, the associated tropic hormones (i.e., gonadotropin releasing hormone, follicle stimulating hormone, and luteinizing hormone) also play a role in fish immunity, indicating an immunomodulating role for the entire hypothalamic-pituitary-gonadal (HPG) axis (reviewed by Segner et al., 2017). Interestingly, there is evidence for bidirectional communication between the HPG axis and immune system, which likely influences how individuals allocate resources to the energetically demanding processes associated with pathogen defense and reproduction.

The specific mechanisms by which sex hormones influence immune function remain elusive, due in part to difficulties in distinguishing the impacts of seasonal alterations in sex-steroid hormone levels on immunity from the seasonal environmental changes that drive changes in both sex-steroid hormone production and immunity. However, there is evidence that estrogens and androgens are capable of directly affecting immune cell function, suggesting that sex-steroid hormones may be a key mediator between seasonal environmental changes and immunity. Specifically, both nuclear and membrane-bound estrogen receptors, as well as nuclear androgen receptors, have been identified in immune tissues (e.g., spleen, head kidney, liver, etc.) and cells (e.g., leukocytes, lymphocytes, etc.) of a variety of teleosts (see Chaves-Pozo et al., 2018, for a review of receptor locations as well as a summary of sex steroid impacts on immunity as determined via in vivo and in vitro studies). In vitro endocrine-disruption studies have provided support for a direct role of estrogens on immune cell activity by showing that estradiol increases the production of pro-inflammatory cytokines, reduces the reactive oxygen species activity, and inhibits the phagocytic cell activity of head kidney cells. Similarly, in vitro studies have shown that both testosterone and 11-ketotestosterone modulate immune gene transcription, reactive oxygen species production, and IgM production. In some cases, similar effects have been observed in in vivo studies; however, because immune cells are subject to influence from a variety of signaling molecules, it is not surprising that the results from in vitro and in vivo studies do not always align. Regardless, investigations aimed at characterizing the impacts of chemically-induced endocrine disruption on immunity in both male and female fish of various reproductive stages are needed to enhance our understanding of how steroid hormone modulate fish immunity.

As is the case with immunocompetence, there are often sex-specific differences in organismal responses to chemical exposures (Gochfeld, 2017). Sex-specific differences are observed in response to endocrine-active compounds, which may result from interactions of these compounds with sex steroid hormone–binding globulins or receptors (Gochfeld, 2017). Moreover, sex-specific responses are also observed in fish following exposures to neurotoxicants, metals, and naturally derived toxins (Rietjens et al., 2018), suggesting that differences in male and female endocrinology cannot account for all sex-specific differences in toxicity. In fact, sex-specific differences may stem from a variety of factors, including genetics, metabolic rate, body size and composition, life history strategies, and even behavior. It is important to note that each of these factors can influence not only the outcome of a chemical exposure, but also the likelihood, magnitude, or route of exposure.

Given that both immunocompetence and responses to chemical exposure may be sex dependent, it is reasonable to assert that males and females may respond differently to immunotoxicants. However, very few fish immunotoxicity studies have directly sought to uncover such sex-specific differences. Of the 241 fish immunotoxicity publications reviewed by Rehberger et al. (2017), 79% did not report the sex of fish used, presenting a major obstacle for inferences regarding differential responses of male and female fish to potential toxicants. Of the fish immunotoxicity studies that have accounted for sex in their experimental designs, evidence suggests that immunotoxic responses, measured at multiple levels of biological organization, are indeed influenced by sex. For instance, on the whole organism level, Thornton et al. (2018) found that brominated diphenyl ether (BDE)-47-exposed male fathead minnows experienced significantly reduced survival rates in response to *Yersinia ruckeri* compared with the BDE-47-exposed females. Moreover, at the cellular level, Hoeger et al. (2005) noted reduced antibody production and lymphocyte numbers in female, but not male, rainbow trout (*Oncorhynchus mykiss*) following exposure to municipal wastewater treatment effluents. At the molecular level, Ye et al. (2012) observed the downregulation of major genes in the complement system in only male marine medaka (*Oryzias melastigma*) following dietary exposure to the flame retardant chemical, BDE-47. The findings of these studies demonstrate the potential for sex-specific differences in immunotoxicity and underscore the need for future studies to consider sex as a confounding factor in immunotoxicity studies.

## Species-specific differences in immunity and immunotoxic responses

**Figure 2.**
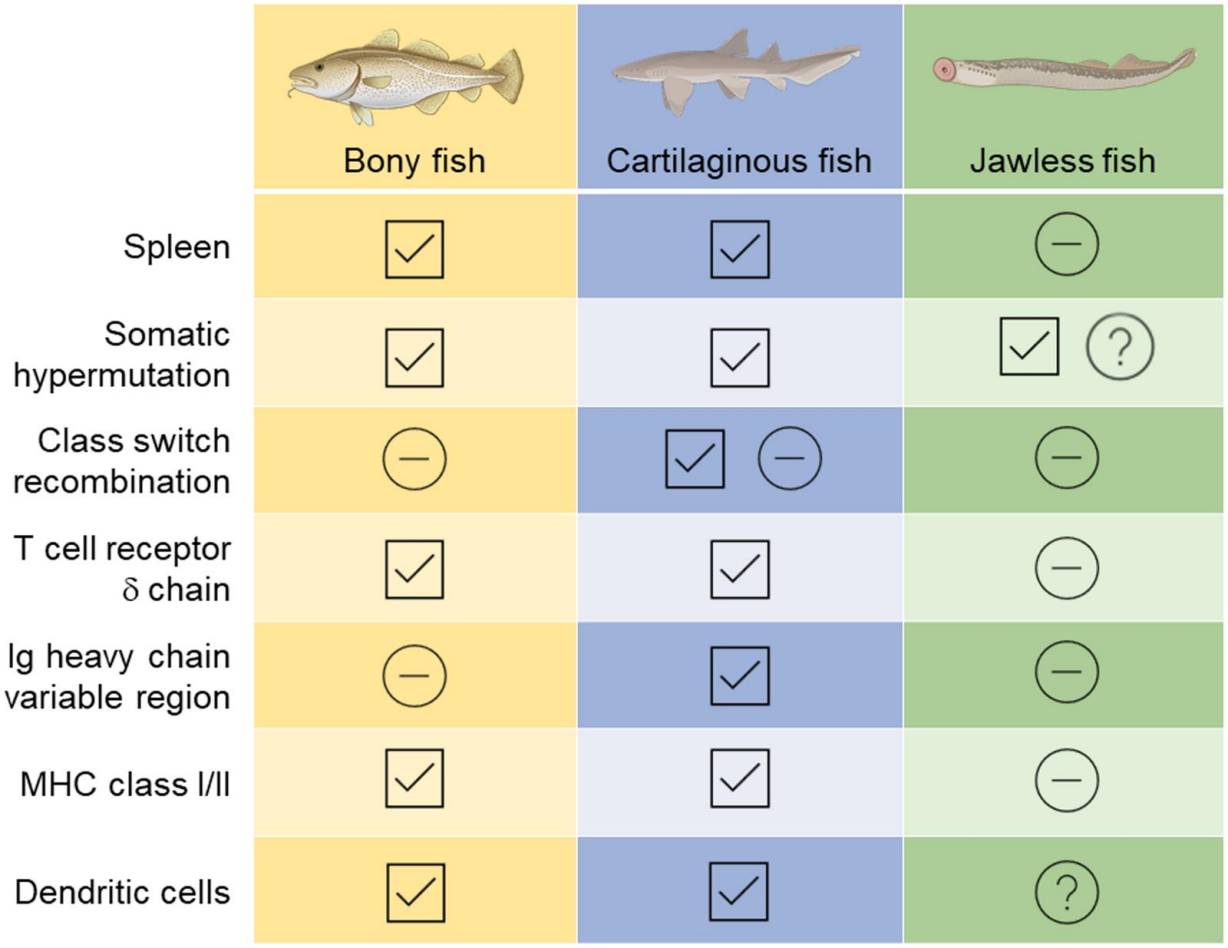
General differences related to adaptive immunity between species representing bony, cartilaginous, and jawless fish. Check marks and minus signs indicate the presence and absence of the immune feature, respectively. Question marks indicate that the presence of the feature has yet to be determined or is inconsistent across species within the class. Figure adapted from Flajnik (2018) and generated using images created with BioRender.com.

With over 34,000 species, fish exhibit a wide array of immune adaptations reflective of their unique ecological niches and evolutionary pressures. One notable aspect of species-specific immune differences lies in the emergence of the adaptive immune system (See Figure 2 for an overview of key differences between bony, cartilaginous, and jawless fish). More primitive jawless fish, such as lampreys and hagfish, pioneered an adaptive immune response relying on variable lymphocyte receptors associated with lymphocyte-like cells for immune defense (Smith et al., 2019). In contrast, more recently evolved jawed fish independently developed adaptive immunity with features resembling higher vertebrates, including the expression of immunoglobulin (Ig) isotypes, T-cell receptors, and major histocompatibility complex (MHC; Smith et al., 2019). Although there is a general recognition that fish species differ in regard to immunity, only a fraction of fish species have been studied in-depth, leaving substantial knowledge gaps regarding baseline immune functions across species as well as their potential susceptibilities to toxicants and pathogens.

The challenge of understanding immunotoxicity responses in fish is compounded by their species diversity and evolutionary life history. For example, cartilaginous and jawed fish have a spleen, whereas jawless fish do not (Flajnik, 2018). Other immune-related organs are also species specific, such as the recently discovered cloacal bursa in salmonids (Løken et al., 2020). Genetic differences among species include the lack of MHC II and an increased number of MHC I genes and Toll-like receptors in Atlantic cod relative to other fish species (Star & Jentoft, 2012). Additionally, in teleost species that temporarily or permanently attach to one another (e.g., anglerfish), genes such as *aicda* and *rag* lose functionality (Swann et al., 2020). As an understanding of fish immunity broadens, additional species-specific differences in the function of immune organs and tissues, such as the naso-pharynx associated lymphoid tissue (Das & Salinas, 2020), are likely to emerge. Further investigation is needed to understand the role of species-specific immune organs, cellular markers, and genes in determining the susceptibility of a given species to immunotoxicants and pathogens.

Evolutionary pressures and ecological adaptations have led to species-specific differences in pathogen-stimulated immune responses. Host-pathogen associations vary among species, affecting the severity of disease caused by familiar or novel pathogens. For instance, Bailey et al. (2019) reported differences in the timing of the adaptive immune response and patterns of tolerance and resistance between brown trout (*Salmo trutta*) and rainbow trout in response to infection by *Tetracapsuloides bryosalmonae*. As the definitive host, brown trout have evolved a host-parasite relationship with *T. bryosalmonae*, enabling a high degree of tolerance. Conversely, rainbow trout, an accidental host species, can clear the parasite completely, but host health is diminished at very low levels of parasite intensity. This demonstrates that even closely related species can vary significantly in their immune response and susceptibility to pathogens.

Understanding species-specific immune function is crucial for assessing the impact of chemical exposures on disease susceptibility. Given the diversity of fish and their immune systems, it is essential to recognize that species may differ in their immunotoxic responses to chemicals. Observing an effect in one species does not necessarily mean the effect will be observed in another. For example, in a comparative study examining the impact of in vitro pesticide exposure on phagocytic cell function and head kidney cell survival, Harford et al. (2005) found a significant decrease in phagocytic activity in rainbowfish (*Melanotaenia fluviatilis*) head kidney isolates at the highest dose of endosulfan (<10 mg/L) tested. Conversely, a significant increase in phagocytic cell activity was observed at the same dose in golden perch (*Macquaria ambigua*) head kidney isolates (Harford et al., 2005). The same study (Harford et al., 2005) observed that chlorpyrifos (10 mg/L) has lymphocytolytic activity in Murray cod (*Maccullochella peelii*) but not in rainbowfish, golden perch, or silver perch (*Bidyanus bidyanus*). Although these findings indicate species-specific differences in response to immunotoxicants, there are few comparative studies on immunotoxicity in fish. Moreover, variability in experimental design makes species comparisons across different studies difficult or impossible.

**Figure 3.**
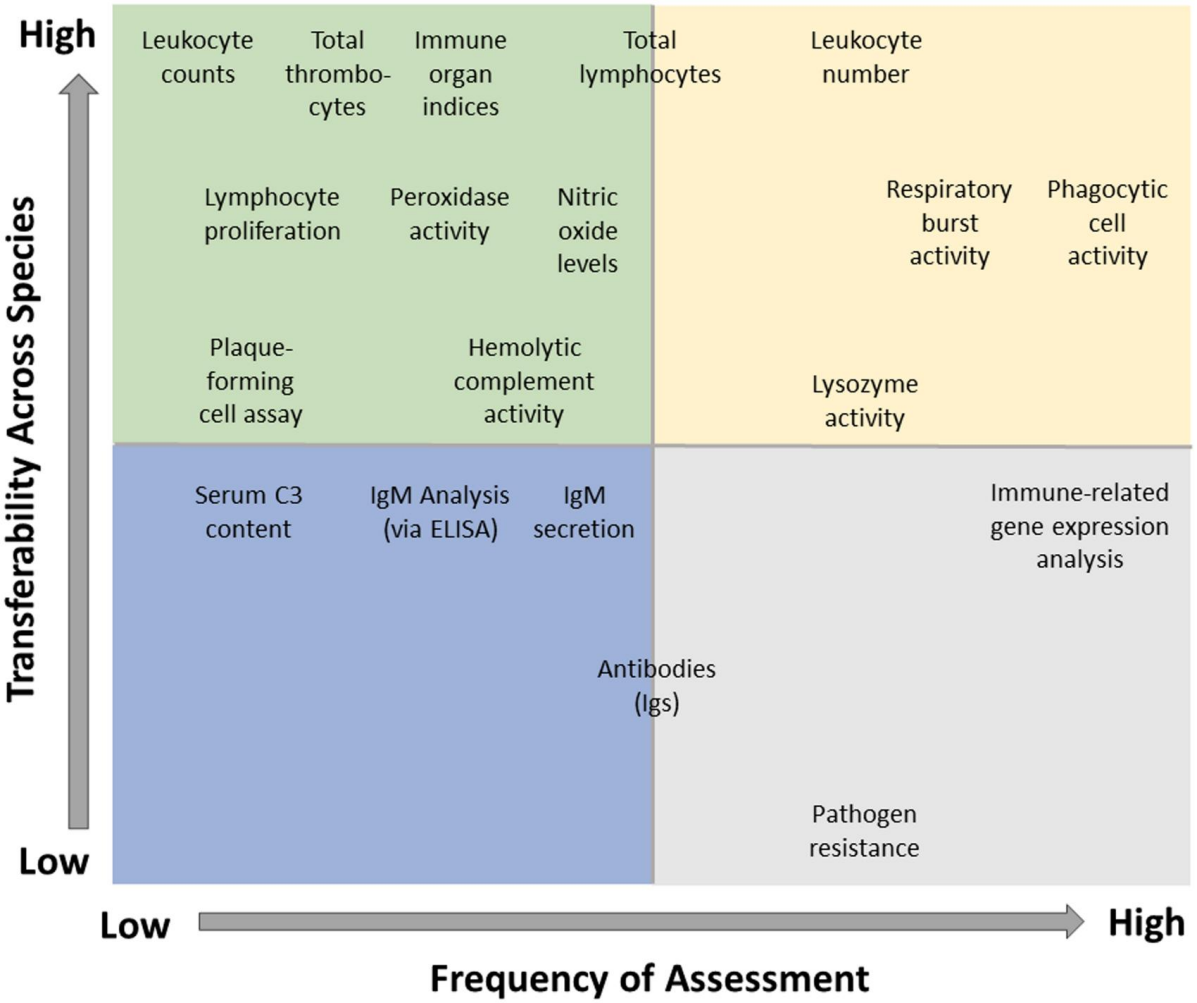
The potential transferability of endpoints across species as well as the frequency with which the most common immunotoxicity-related endpoints are measured (as reported in Rehberger et al., 2017).

Evaluating and comparing immunotoxicity in fish species is constrained by the limited availability of standardized assays and methods outside of a few key species. A review by Rehberger et al. (2017) revealed four families dominating publications on fish immunotoxicity (Salmonidae > Cyprinidae = Sparidae > Cichlidae), with rainbow trout dominating at the species level. As such, a variety of endpoints have been used to evaluate immunotoxicity in rainbow trout. The extent to which these endpoints and associated methods of evaluation are transferable to other species is dependent on the nature of the endpoint itself and the species in which the endpoint will be measured (See Figure 3 for a summary of frequently measured endpoints and their potential cross-species transferability). For example, ex vivo assays that enable assessment of phagocytic cell activity and respiratory burst are typically transferable between species, whereas genomic resources (i.e., primer sequences) that enable quantification of immune gene expression may only be transferrable to closely-related species. In contrast, pathogen resistance assays in which fish survival is monitored following infection with a pathogen tends to be species specific due to specificity in host-pathogen pairings. Development and standardization of methods that can be applied across a multitude of species is a daunting but crucial task for the field of immunotoxicology and future application of such methods in risk assessment. Establishing common endpoints in immunotoxicity testing will allow for meaningful comparisons across species and may be useful for identifying the mechanisms underlying immunotoxicity (Segner et al., 2022). As aquatic immunotoxicity research expands, it will be essential to explore and characterize the immune systems of a broader range of fish species, taking into careful consideration their unique ecological niches, genetic makeup, and evolutionary adaptations. This knowledge will contribute to a more comprehensive understanding of fish health and aid in the development of targeted conservation and management strategies.

## Conclusions and future research directions

The immune system of fish, consisting of both innate and adaptive components, is sensitive to toxicants. Environmental stressors and chemicals can cause immunomodulation, which can lead to reduced pathogen resistance, death, population declines, alterations in ecosystem function, and severe economic consequences. Factors such as life stage, sex, and species, influence fish immunity and immunotoxic responses in both laboratory and field-based studies. However, these factors remain understudied, leaving substantial gaps in our ability to adequately design experiments that account for these potential confounding factors, interpret data, translate findings into broader contexts, and carry out robust environmental risk assessments. To advance fish immunotoxicity studies, it is critical to further explore and better understand these sources of variation. With regard to life stage, laboratory studies aimed at identifying critical windows of sensitivity for the development of organizational effects that lead to permanent alterations in immunity and disease resistance are needed. Such studies would help identify vulnerable life stages, which is a key consideration when determining appropriate targets for ecological risk assessments. With regard to sex, direct comparison of male and female immune responses to pathogens in both the presence and absence of chemical contaminants are needed. Such studies should include robust assessments of HPG axis and reproductive parameters to advance our understanding of the mechanisms underlying sex-specific differences in immunity and immunotoxicity. Finally, comparative immunotoxicological studies in which direct comparisons of immune function and immunotoxic responses are characterized in multiple species are needed to identify not only vulnerable taxa but also to identify species that can serve as appropriate surrogates in immunotoxicity assessments.

The sources of variation addressed in this article represent only a fraction of the biotic factors underscoring the complexity of the immune system and its responses to chemicals. Thus, it is important to recognize the intricate interplay of additional factors that may also affect immune outcomes. New dimensions to consider in the field of immunotoxicology include the influence of behavior, diet, microbiome, and epigenetic modifications. Behavioral phenotypes were the focus of a recent study demonstrating differences in the pathogen-stimulated immune responses of bold versus shy perch (*Perca fluviatilis*; Gebauer et al., 2023). Likewise, nutritional factors have also been shown to affect overall immune responses and health of smallmouth bass (*Micropterus dolomieu*) as evidenced by differences in leukocyte bactericidal activity and mitogen responses between bass fed artificial pelleted diets and those on live feed (Ottinger et al., 2019). The fish microbiome has also been implicated as having an impact on immunity as indicated by a study showing that alterations in the microbiome of larval zebrafish affect their survival following infection with *Vibrio anguillarum* (Vargas et al., 2021). Epigenetic modifications may also alter immunological responses in fish as demonstrated by an increase in reovirus susceptibility grass carp (*Ctenopharyngodon idella*) with epigenetic modifications (Shang et al., 2016). The variety of factors that influence immune function and immune system responses to chemicals in fish highlight the multifaceted and complex nature of immunotoxicity assessments in fish. Future research could focus not only on characterizing the impacts of biological factors on fish immunity but also on developing methods and approaches for immunotoxicity assessments that adequately account for these potential confounding factors so that we can gain a clearer understanding of mechanisms and consequences of chemical-induced immunotoxicity in fish.

## Data Availability

Data will be made available on request to the corresponding author (m.jeffries@tcu.edu).
